# Antenatal urolithiasis: A case report

**DOI:** 10.3389/fped.2022.933948

**Published:** 2022-08-11

**Authors:** Sumona Bose, Arpana Iyengar, Attibele Mahadevaiah Shubha

**Affiliations:** ^1^St. John’s National Academy of Health Sciences, Bengaluru, India; ^2^Department of Pediatric Surgery, Bengaluru, India; ^3^Department of Pediatric Nephrology, Bengaluru, India

**Keywords:** antenatal, obstructive calculus, hydronephrosis, surveillance, surgery

## Abstract

**Introduction:**

Although pediatric urolithiasis is an established entity, its antenatal diagnosis is rare. We hereby report a case detected at 20 weeks gestation and discuss the etiopathogenesis, predisposition, and surveillance following intervention.

**Case report:**

A 2-year-old girl with left renal pelvic calculus detected antenatally at 20 weeks was evaluated. Left hydronephrosis, obstructive pelvic calculus with a decrease in differential renal function on ethylene dicysteine (EC) renogram was confirmed. The metabolic workup was normal. Following stone extraction by left pyelolithotomy, a left ureteropelvic junction obstruction secondary to a mucosal valve was apparent which was excised and left pyeloplasty was done. Stone analysis revealed 100% cystine. Differential renal function and drainage improved post-surgery. The child, however, did not have a follow-up in the interim and presented with a recurrent stone one and a half years later.

**Conclusion:**

Knowledge of antenatal urolithiasis ensures continued follow-up, evaluation for metabolic disorders, and associated structural defects, especially with increasing stone size and increasing hydronephrosis. This helps in timely intervention and continued surveillance.

## Introduction

Although 20–30% of all the prenatally identified anomalies relate to the urinary tract (1), antenatal diagnosis of urolithiasis is rare. Only two cases have been documented in the literature to date. Here, we report the management of a child with antenatal urolithiasis and discuss the etiopathogenesis, predisposition, and follow-up; along with a brief review of literature on Cystinuria.

## Case report

**FIGURE 1:**
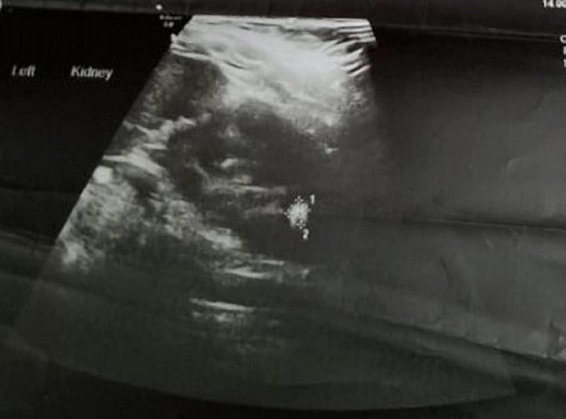
Renal scan showing left renal pelvis of 13 mm with a 2 cm pelvic stone.

**FIGURE 2:**
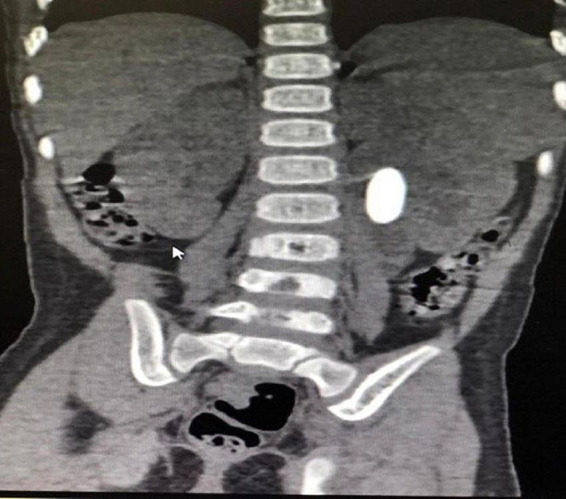
CT KUB showing hydronephrotic left kidney (PD-13.1 mm), with 2.4 cm × 1.1 cm oval calculus, normal ureter.

A 2-year-old asymptomatic girl, was referred for the management of left renal calculus. Antenatal ultrasound at 20 weeks gestation had multiple echogenic foci in the left lower calyx which persisted at 28 weeks with normal amniotic fluid volume. She was born full-term, weighed 2.8 kg, and had an uneventful perinatal period. Renal ultrasound at 6 months showed 2–3 hyperechoic foci in the lower pole of the left kidney, with the largest being 6.2 mm with a pelvic diameter of 9 mm, spot urine calcium (8.1 mg/dl), urine creatinine (17.59 mg/dl), and calcium/creatinine ratio of 0.46. At 1 year of age, serum creatinine was 0.38 mg/dl (0.6–0.9 mg/dl). Serum calcium (10.8 mg/dl) (normal range 9–11 mg/dl), phosphorous (6.6 mg/dl) (normal range 4–7 mg/dl), magnesium (2.5 mg/dl) (normal range 1.8–2.4 mg/dl), alkaline phosphatase (222 U/L) (81–350 U/L), and uric acid (3.5 mg/dl) (2.5–5.5 mg/dl) were normal. She was referred to us for further management due to a progressive increase in the size of the calcific foci with increasing hydronephrosis. At 2 years, she weighed 9.3 kg and 81 cm in height, with a blood pressure of 100/70 mmHg (99 percentile). Her hemoglobin, electrolytes, serum bicarbonate, and renal functions were normal but eGFR was 73 ml/min- (> 90 ml/min being normal). Ultrasound showed a left renal pelvis diameter of 13 mm with a 2 cm stone in the pelvis (Figure 1). The right kidney was normal. Computerized tomography (CT) revealed a hydronephrotic (PD-13.1 mm) left kidney with 2.4/1.1 cm oval calculus and a normal ureter (Figure 2). Ethylene dicysteine renogram confirmed obstruction of the left kidney with differential renal function (DRF) of 33% and 67% for the right kidney. There was no history of stone disease in the family.

**FIGURE 3:**
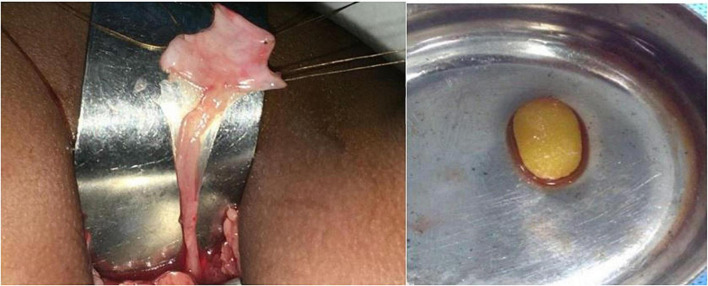
Intra-operative picture showing valve causing narrowing of the ureteropelvic junction with retrieved calculus.

**FIGURE 4:**
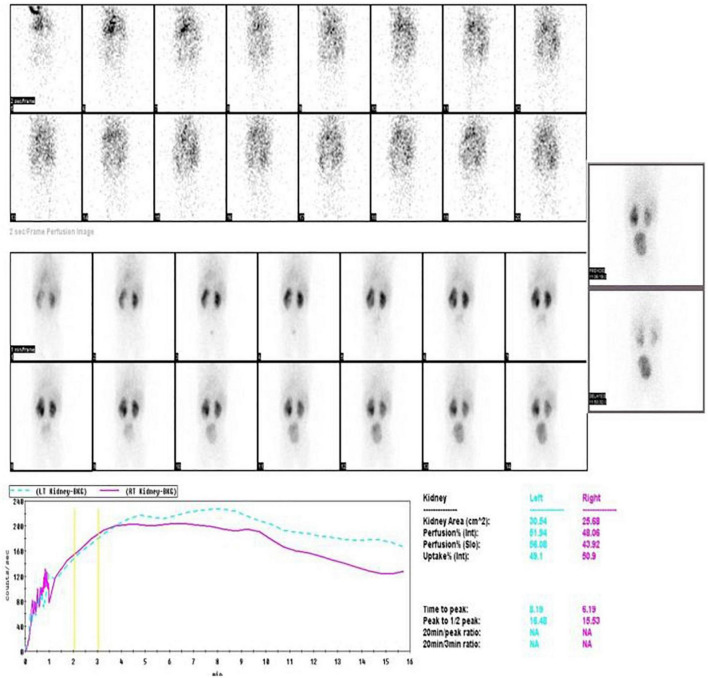
Post-operative EC scan after 3 months showing resolution of hydronephrosis, improvement in drainage, and DRF of 49%.

In view of the large size stone, endoscopic management was deferred. A 3 cm × 3 cm calculus, partially intra- renal extending into the lower calyx was retrieved by a left pyelolithotomy. On probing the ureter, a mucosal valve causing narrowing at the ureteropelvic junction was noted (Figure 3). This part of the narrow segment was excised and pyeloplasty was done over a 4 Fr, 16 cm DJ stent which was removed after 3 weeks. She had an uneventful recovery. The stone weighed 2.58 g and contained 100% cystine. Postoperatively, her serum creatinine was 0.4 mg/dl, bicarbonate was 27.6 mmol/L, and eGFR was 82%. Ethylene dicysteine renogram after 3 months showed resolution of left HDN, good drainage, and an increase in DRF to 49% (Figure 4). She was advised to increase fluid intake, a salt-restricted diet, and regular follow-up which she defaulted. She presented one and a half years later with 7 mm (lower pole) and 9 mm (pelvic) recurrent non-obstructive stones in the left kidney. She is presently on potrate and D-penicillamine.

## Discussion

Incidence of urolithiasis is between 1 in 3,000 and 1 in 8,000 of all pediatric admissions and is 10% of that in adults (2). Pediatric stone disease is more common in the male child and noted at 6–7 years of age with a slightly higher incidence in the first 2 years (3). However, to date only two cases of prenatal urolithiasis have been documented, the present one being the third.

The first case was a 28-week monochorionic, diamniotic twin gestation, and mild polyhydramnios with one of the twins having a 3-mm echogenic focus in the upper part of the right kidney. At 3 month postnatal ultrasound, it was revealed that there were multiple right upper pole stones with the largest measuring 5 mm. The baby was asymptomatic with normal metabolic workup and VCUG and was well up to 6 months follow-up (4).

Rhodes et al. reported antenatal urolithiasis detected at 34 weeks gestation as an echogenic focus in the upper pole of the right kidney which persisted up to 4 months. No metabolic or structural abnormalities were noted in the child during follow-up (5). Detection of renal stone at 20 weeks gestation as in ours is the earliest reported case to date. Occurrence in the female child and occurrence on the left side are exceptional.

Urinary stones are composed of lithogenic crystal agglomerations and are formed on the renal papillae by adherence to the damaged renal epithelium. The imbalance of the activating factors such as high calcium/oxalate and the inhibitory factors such as low citrate excretion dictates stone formation. Almost 50% of pediatric urolithiasis cases are idiopathic, and 30 and 20% are due to hypercalciuria and hyperoxaluria, respectively. Rarely hyperuricosuria, xanthinuria, and hypocitruria are the cause (6). Almost one-third may have structural abnormalities, and few are associated with renal foreign body, papillary necrosis, or Urinary tract infections with urease-producing organisms (7). Urolithiasis is often detected as hyperechoic foci on ultrasound and confirmed by a CT scan. Spontaneous resolution is less likely, and these children, when symptomatic, may need operative intervention (8). Stones less than 5 mm in size are known to pass spontaneously.

A 2-day-old male neonate with gross hematuria and a 3–4 mm calculus in the upper pole of the right kidney with no metabolic or structural abnormality was managed conservatively (9), whereas a series of eight premature infants with persistent renal stones were successfully treated by extracorporeal shock wave lithotripsy at the mean age of 13 months and mean body weight of 7.7 kg (10).

As in our child, who was asymptomatic but had persistence of stone with a gradual increase in size and normal metabolic parameters, a thorough evaluation for a structural abnormality is stressed. Following stone retrieval, probing of the ureter for patency is also emphasized so that an occult obstructive pathology is not missed. In the present case, the valve seems to be a result of mucosal inflammation caused by the stone present since the prenatal period. A 100% cystine on stone analysis confirms Cystinuria.

Cystinuria is an autosomal recessive disorder, characterized by failure of reabsorption of cystine and other dibasic amino acids in the renal tubules (11). Cystinuria accounts for 6–10% of pediatric urolithiasis with a prevalence of 1 in 7,000 live births (6). SLC3A1 and SLC7A9 genes are established as causative factors (12). Detection of a hyperechogenic colon in antenatal scans suggests cystinuria and has a high positive predictive value (11). Fetal amniotic fluid contains high amount of cystine excreted by fetal kidneys which when swallowed by the baby results in a high amount of cystine in the colon causing hyper-echogenicity on ultrasound (13).

Amat S. et al. concluded that the presence of a hyperechoic colon at a routine ultrasound scan before 36 weeks gestation should prompt screening for cystinuria at birth, while later observation (> 36 weeks) does not relate to any disease (14). In our patient, colonic hyperechogenicity was not noted in the antenatal ultrasound. Cystinuria is diagnosed by stone analysis (X-ray diffraction/infra-red spectroscopy), observation of hexagonal cystine crystals in the urinary sediment of first-morning urine, or detection of cystine and dibasic amino acids in urine. Plain CT KUB has sensitivity and specificity of 98 and 100%, respectively, for detecting stones (15). Assessment and surveillance of recurrent stone formers are done using ultrasound to minimize cumulative radiation exposure. Conservative treatment includes adequate hydration, a salt-restricted diet, and alkalinization with the addition of thiol derivatives in refractory cases. Renal decompression (stent/nephrostomy) is done in infected/obstructed kidneys. Small renal/ureteric stones are observed. Larger stones > 2 cm with pain, infection, hematuria, and stone growth are treated surgically/PCNL. Stones < 2 cm can be treated with flexible ureteroscopy, ESWL, or miniaturized PCNL (16). However, patients with cystinuria have a higher risk of chronic kidney disease and early onset hypertension and therefore need regular follow-up (11).

## Conclusion

The current case is one of the rare occurrences of prenatal urolithiasis thus far reported in the literature. Knowledge of antenatal urolithiasis helps in planning the most appropriate treatment. Emphasis is on prolonged follow-up and evaluation for associated structural defects and metabolic disorders, especially in cases with increasing stone size and increasing hydronephrosis. This will prompt a timely surgical intervention, initiation of appropriate medical treatment, and regular follow-up, all aiming to preserve renal function.

## Data availability statement

The raw data supporting the conclusions of this article will be made available by the authors, without undue reservation.

## Ethics statement

The studies involving human participants were reviewed and approved by the Institutional Ethics Committee, St. Johns Medical College and Hospital, Bangalore. Written informed consent to participate in this study was provided by the participants’ legal guardian/next of kin. Written informed consent was obtained from the individual(s), and minor(s)’ legal guardian/next of kin, for the publication of any potentially identifiable images or data included in this article.

## Author contributions

SB contributed towards conception and design, data collation, analysis and interpretation of data, drafting of the manuscript, and involved in obtaining ethical approval. AS and AI contributed toward study design, patient management, data collation, drafting, critical revision and editing of the manuscript, and supervision at all levels. All authors have reviewed and approved the final manuscript.

## Conflict of interest

The authors declare that the research was conducted in the absence of any commercial or financial relationships that could be construed as a potential conflict of interest.

## Publisher’s note

All claims expressed in this article are solely those of the authors and do not necessarily represent those of their affiliated organizations, or those of the publisher, the editors and the reviewers. Any product that may be evaluated in this article, or claim that may be made by its manufacturer, is not guaranteed or endorsed by the publisher.

## Abbreviations

EC: ethylene dicysteine
eGFR: estimated glomerular filtration rate
PD: pelvic diameter
DRF: Differential renal function
HDN: hydronephrosis.
